# Genomic analysis reveals selection pressure and the emergence of molecular insecticide resistance in the malaria vector, *Anopheles gambiae*, from Western Ethiopia

**DOI:** 10.1186/s13071-026-07582-z

**Published:** 2026-08-05

**Authors:** Fekadu Gemechu, Kelly L. Bennett, Sisay Dugassa, Anastasia Hernandez-Koutoucheva, Araya Eukubay, Alistair Miles, Deriba Abera, Chris S. Clarkson, Lemu Golassa

**Affiliations:** 1 Ethiopian Public Health Institute, Addis Ababa, Ethiopia; 2 Center for Pathobiology, Aklilu Lemma Institute of Health Research, Addis Ababa University, Addis Ababa, Ethiopia; 3 Liverpool School of Tropical Medicine, Pembroke Place, Liverpool, L3 5QA UK; 4 Department of Zoological Sciences, Addis Ababa University, Addis Ababa, Ethiopia; 5 Ellison Institute of Technology, Littlemore, Oxford, OX4 4GE UK

**Keywords:** *Anopheles gambiae* sensu stricto, Target-site insecticide resistance, Metabolic insecticide resistance, Selection scans, Ethiopia, Whole-genome sequencing, Malaria vector

## Abstract

**Background:**

Insecticide resistance poses a significant challenge to malaria control, driven by diverse molecular mechanisms, whose distribution remains poorly characterized in Ethiopia. This study presents the first results using whole-genome sequence data of *Anopheles gambiae* from Ethiopia, confirming its presence in the western region of the country and expanding its known geographical distribution.

**Methods:**

Analysis of single-nucleotide polymorphisms and copy number variants focussed on key target-site insecticide resistance genes, including the voltage-gated sodium channel (*Vgsc*), acetylcholinesterase-1 (*Ace-1*), the gamma-aminobutyric acid (*GABA*)-gated chloride channel (*Rdl*) gene, as well as metabolic resistance loci such as cytochrome P450s (*Cyp6m2, Cyp6aa/p*, *Cyp9k1*) and carboxylesterases (*Coeae2f*, *Coeae2–7g*).

**Results:**

Genomic analysis revealed high frequencies of *Vgsc*-L995F (kdr-west) mutations, alongside amplifications at *Cyp6aa/p*, *Cyp9k1*, and *Gste2*. Notably, frequencies of *Vgsc* and *Gste2* variants exhibited differences on a local scale, while *Vgsc* and *Cyp9k1* variant frequencies also fluctuated seasonally. Findings highlight the need for site-specific monitoring on a fine temporal scale. Furthermore, genome-wide selection scans using phased haplotypes identified emerging signals of selection at loci with a potential link to insecticide resistance, including a signal spanning 2L:33,767,545–34,391,778, which expands the catalogue of candidate loci for functional validation.

**Conclusions:**

Together, the presence of high frequencies of known insecticide resistance markers suggests that *An*. *gambiae* populations may be largely refractory to pyrethroids and moderately resistant to organophosphate (OPs) insecticides in western Ethiopia. Findings necessitate a better understanding of the *An. gambiae* geographical distribution in Ethiopia, accompanied by resistance-informed malaria control interventions targeting the vector.

**Graphical Abstract:**

**Figure:**
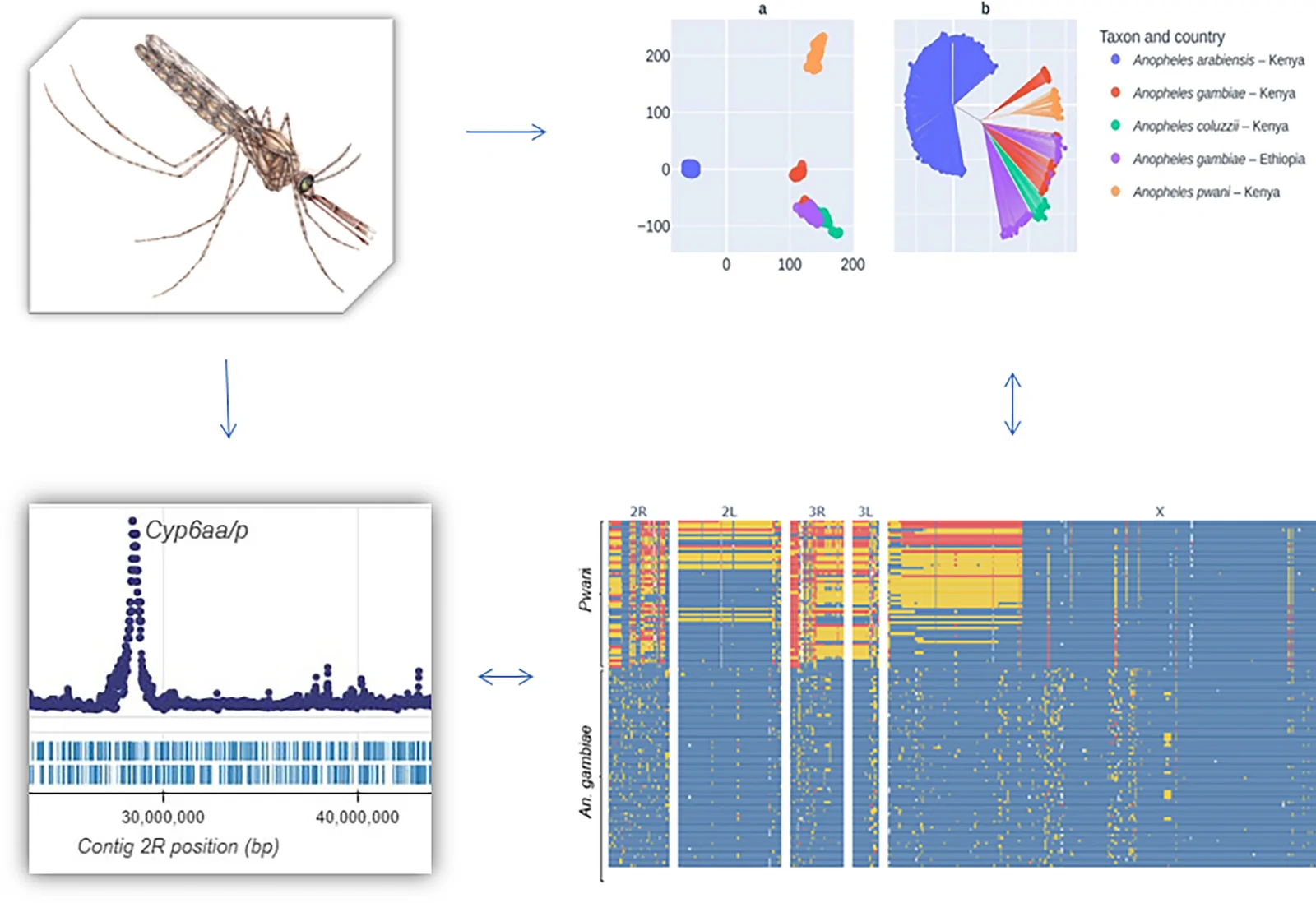

**Supplementary Information:**

The online version contains supplementary material available at https://doi.org/10.1186/s13071-026-07582-z.

## Background

From a global perspective, insecticide resistance poses a significant challenge to malaria control [1, 2], and a range of molecular mechanisms mediates it. Major mechanisms of insecticide resistance include target-site resistance, in which modification of the target site occurs through changes in the structure of the insect’s proteins, and metabolic resistance, in which increased enzyme production and/or efficiency enhance the detoxification of insecticides [3]. Furthermore, insecticide resistance can be promoted by reduced cuticle penetration or changes in behaviour, such as avoiding areas treated with insecticides or through alterations in feeding habits [4–6]. Due to their importance in guiding malaria control, the insecticide resistance phenotypes of its major vectors, *Anopheles* mosquitoes, are routinely tracked using bioassays, often without identifying their underlying genomic determinants [3, 5]. Recently, however, there have been calls to increase the use of molecular assays to determine the genomic mechanism of resistance, to guide effective use of interventions, and to track the impact of their implementation [7–9]. Even so, because molecular assays typically focus on a few known genomic variants, other novel markers rising in frequency and under selection pressure from insecticides can be missed [10, 11]. This is particularly true because *Anopheles* mosquitoes rapidly evolve novel mechanisms of defence [12, 13]. The use of whole-genome sequencing offers the advantage of identifying novel genes under positive selection, potentially linked to insecticide resistance. Such an approach can bridge the existing gaps in prioritizing genes for validation studies or identifying markers for longitudinal tracking [14–16].

In *Anopheles* mosquitoes, the major genes involved in target-site resistance are the voltage-gated sodium channel (*Vgsc*), acetylcholinesterase (*Ace-1*), and the gamma-aminobutyric acid (*GABA*)-gated chloride channel (*Rdl*). These genes are associated with resistance to pyrethroids and DDT [17, 18], organophosphates [19, 20], and carbamates [21–23], respectively. Although primarily associated with resistance to dieldrin, the *Rdl* locus is also the target of Fipronil and a secondary target for neonicotinoids and pyrethroids [24–26]. In addition, metabolic insecticide resistance is frequently driven by copy number variants (CNVs), particularly at cytochrome P450 genes such as the *Cyp6aa*/*p* [21, 23] or *Cyp6m2-z1* gene clusters, *Cyp9k1* [22], carboxylesterases such as *Coeae2f* [23, 27, 28], *Coeae2-7 g* [23], as well as the glutathione S-transferase *Gste2* [29, 30]. These gene variants often emerge due to selective pressures from insecticide-based interventions, such as indoor residual spraying (IRS) and long-lasting insecticidal nets (LLINs) of organophosphates [19, 31, 32]. Despite the increasing emergence of resistance, insecticide-based tools remain the primary method for malaria control interventions, including in Ethiopia [33].

In Ethiopia, major malaria vectors are members of the *Anopheles gambiae* species complex, including *Anopheles arabiensis*, *Anopheles quadriannulatus,* and the *Anopheles funestus* species group [34–36]. Additionally, another major malaria vector in Africa, *Anopheles gambiae s. s.,* has been reported from the Meskan and Sodo districts of the Gurage zone, south-central Ethiopia [37]. Since members of the *Anopheles gambiae* complex are morphologically indistinguishable [38–41], they were molecularly identified based on species-specific single-nucleotide polymorphism (SNPs) in the intergenic spacer region (IGS) using polymerase chain reaction (PCR) [42]. Furthermore, PCR-based approaches have limited resolution and sensitivity, and may be subject to primer failure or misidentification due to genome polymorphism [43–45]. However, *An. gambiae* is present in other neighbouring East African countries, including Kenya [46] and Sudan [47], while Ethiopia harbours a suitable habitat for *An. gambiae* [48], including wet-humid climatic conditions typical of its distribution [36, 49]. Studies from nearby East Africa have shown that *An. gambiae* has evolved insecticide resistance, including target-site resistance through the L995F and L995S substitutions at *Vgsc* and the G119S substitution at *Ace-1* [50, 51], as well as metabolic resistance due to copy number variations (CNVs) at cytochrome P450 loci, including *Cyp6m2*, *Cyp6aa1,* and *Cyp9k1* [22, 52, 53]. Additionally, elevated expression and gene amplification of carboxylesterases have been implicated in insecticide resistance, particularly at major loci such as *Coeae2f* and *Coeae6o,* which play a role in detoxifying OPs and pyrethroid resistance [23, 27]. However, since *Anopheles gambiae* has not yet been confirmed in Ethiopia, there has been no report of insecticide resistance or its molecular markers in the country. An understanding of its distribution and the extent of insecticide resistance would aid effective intervention against a recent malaria resurgence in the country [54–56].

Our study presents the first confirmation of *Anopheles gambiae* presence in Ethiopia using whole-genome sequencing. We determined the presence and frequency of known insecticide resistance markers based on single-nucleotide polymorphism (SNP) and copy number variant (CNV) data. To access recent adaptive changes, we performed a genome-wide selection scan (GWSS) using phased haplotypes to investigate evidence of novel markers under selection from insecticides.

## Methods

### The study areas

**Fig. 1:**
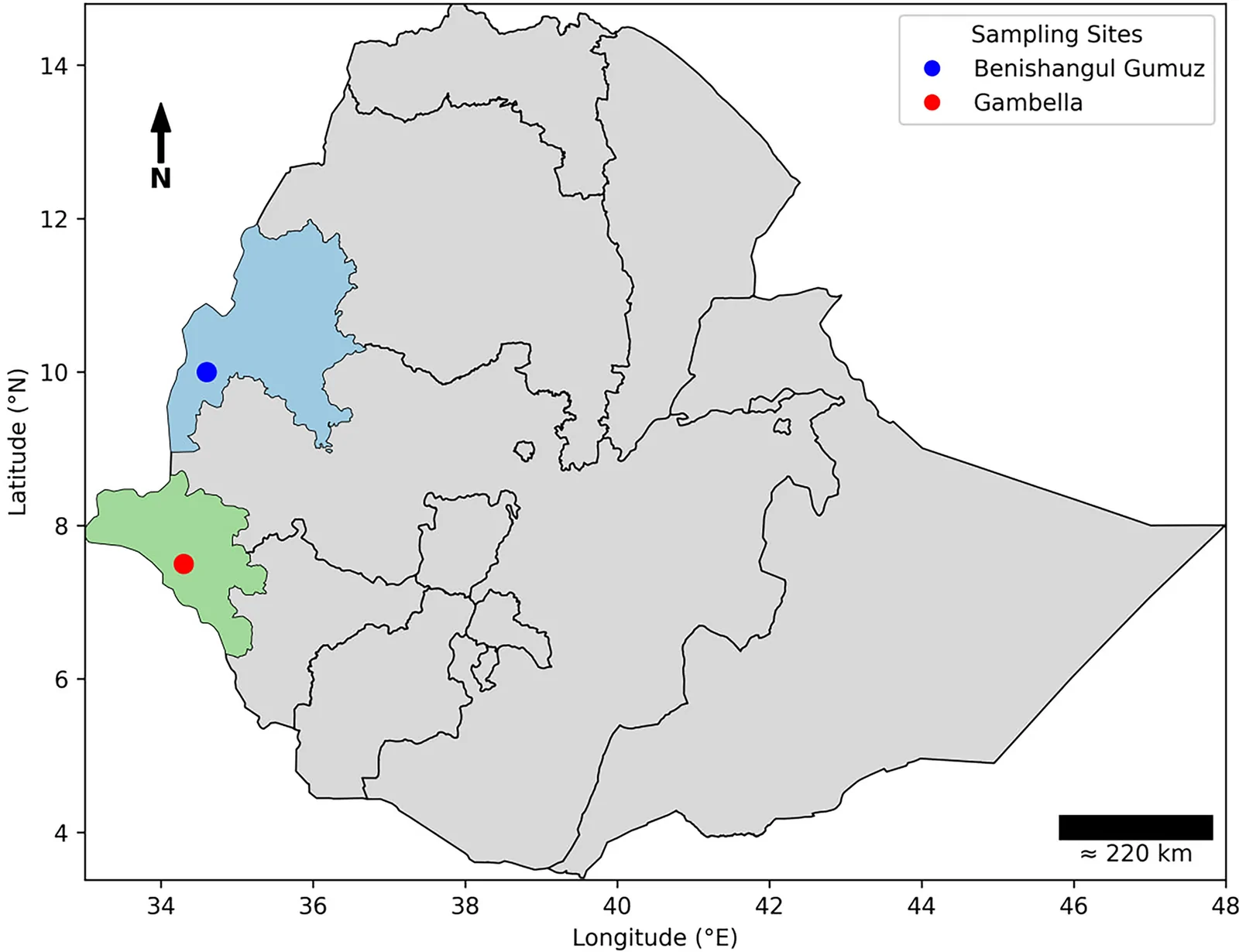
The map of the study sites from which mosquitoes were collected. The administrative regions are outlined, with the blue point representing Benishangul-Gumuz and the red point representing Gambella

*Anopheles mosquitoes* were collected from the border region of western Ethiopia, including sites in Gambella and Benishangul-Gumuz in the districts of Agnuak and Kurmuk, respectively (Table 1, Fig. 1). The areas were characterized by a mixed ecological setting, including seasonally flooded lowlands, riverine and swampy habitats, irrigated agricultural fields, and peridomestic environments near human dwellings.

**Table 1:** Number of *Anopheles* mosquito processed by whole-genome sequencing from each location and season

Sample location (region)	Year of collection	*Anopheles gambiae* sensu stricto sequences		
		Minor season	Major season	Total sample passed sequence QC
Gambella	2023	16	15	31
Benishangul-Gumuz	2023	12	36	48

Such habitat diversity supports year-round mosquito breeding and contributes to persistent malaria transmission [57, 58]. Collections were performed in 2023, during both the major and minor malaria transmission seasons in Ethiopia. The major transmission season typically occurs from September to December, following the main rainy season (June–September) when extensive rainfall creates abundant breeding habitats for mosquitoes. The minor transmission season spans April to June, following a short rainy season (February to April).

### Mosquito collections

Both adult and immature mosquitoes reared to adulthood were collected and identified as *Anopheles gambiae s. l.* using a morphological identification key [59]. Adults were collected using a standard Prokopack aspirator and manual aspiration (CDC) from both indoor and outdoor areas of residential areas. CDC light traps were also deployed to collect host-seeking adult mosquitoes [60]. Immature mosquitoes were collected using standard dippers (CDC) from a variety of breeding habitats, including sunlit puddles, rain-filled hoofprints, irrigation channels, and temporary pools near human dwellings, which were intensively searched and sampled during each surveillance round. Individual mosquitoes were preserved in absolute ethanol in PCR plates for DNA extraction and subsequent sequencing. DNA was extracted from individual mosquitoes using the Qiagen DNeasy Blood and Tissue Kit (Qiagen Sciences, MD, USA), following the manufacturer’s protocol.

### Whole-genome sequencing

Mosquitoes were prepared for whole-genome sequencing according to the guidelines of the *Anopheles gambiae* 1000 Genomes Project [61, 62], which involved generating paired-end multiplex libraries using the Illumina protocol. However, instead of nebulization, genomic DNA was fragmented using Covaris Adaptive Focused Acoustics. Multiplexes comprised 12 tagged individuals of mosquitoes, and 150 bp paired-end reads were generated using an Illumina NovaSeq sequencer.

### Sequencing alignment and variant processing

Sequencing reads were aligned to the AgamP4 reference genome using BWA-mem (Burrows-Wheeler Aligner) [63–65]. Single-nucleotide polymorphism (SNP) data were generated using GATK version 3.7.0 [66] according to the protocols of the *Anopheles gambiae* 1000 Genomes Project [61]. Samples with a median coverage less than 10X, with less than 50% genome coverage, or with a high contamination threshold (> 4.5%) were excluded from further analysis. Biallelic SNPs passing site filters were phased into haplotypes using a combination of read-backed phasing with WhatsHap V1.0 [67, 68] and statistical phasing with SHAPEIT V4.2 [69]. Copy number variants (CNVs) were called following the procedures described in Lucas et al. (2019) using a Gaussian Hidden Markov Model (HMM) to calculate the copy number for each sample across 300 base pair windows of the genome using normalized coverage data. CNV calls were filtered for those with a high likelihood > 1000 predicted by the HMM model. To increase CNV prediction accuracy, individuals with high coverage variance (> 0.35) were excluded. Full specifications for the alignment and variant processing pipelines are accessible in the MalariaGen/pipelines GitHub repository. The frequency of CNVs was called at metabolic resistance loci on a per-locus basis considering individuals with a copy number > 2 or > 1 for the X chromosome in males [70].

## Results

### Taxonomic structure analysis

We sought to investigate taxonomic status using a coordinated panel of ancestry-informative markers (AIMs) developed during the *Anopheles gambiae* 1000 Genomes (Ag1000G) project (2017), which includes SNPs highly differentiated among *An. arabiensis*, *An. gambiae* and *An. coluzzii* [71]. First, a total of 2612 AIM variants were employed to determine the taxonomic status of *An. gambiae / An. coluzzii* from the closely related and morphologically identical species *Anopheles arabiensis.* Analysis revealed that all samples had an AIM profile typical of either *Anopheles gambiae* or *An. coluzzii* (Figure S1- a). Sequentially, a total of 700 AIM variants were then used to distinguish *An. gambiae* from its sister species *An. coluzzii.* It was further confirmed that all samples had an AIM profile characteristic of *An. gambiae* (Fig. 1b).

**Fig. 2:**
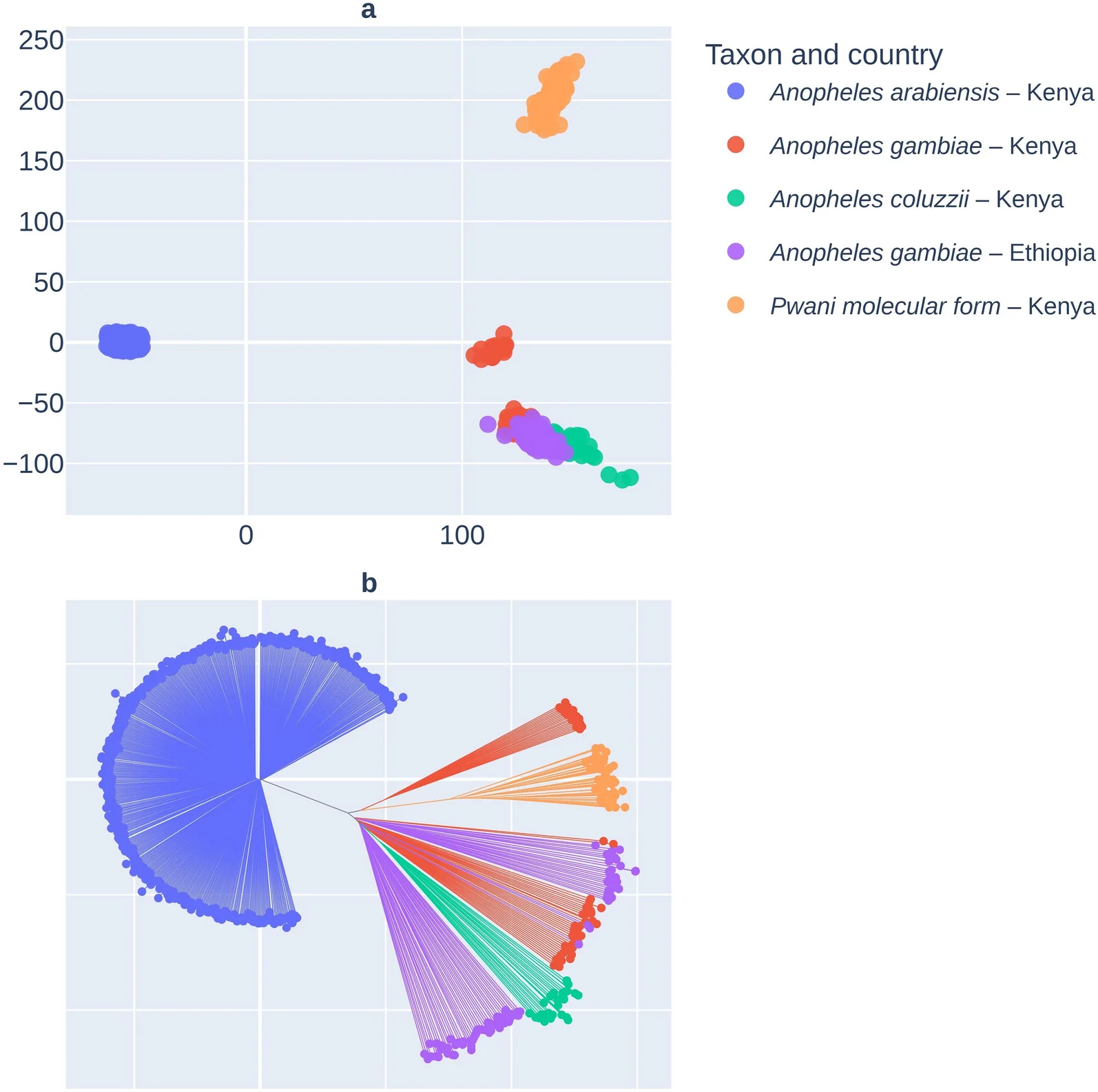
The taxonomic structure of the *Anopheles gambiae* complex in East Africa: **a** Principal component analysis (PCA) of biallelic SNPs on the 3L chromosome for individuals classified as *An. gambiae s. s., An. coluzzii, An. arabiensis* and *An. gambiae pwani* molecular form. Each point represents an individual mosquito, coloured by taxon assignment, with PC1 and PC2 capturing major axes of genetic variation. **b** Neighbour-joining tree constructed from pairwise genetic distances among individuals confirms taxonomic relationships and divergence within the complex. Branch lengths reflect genetic distance

Since the AIMs used in analysis are based on a limited number of genomic markers from individuals representing a restricted geographical distribution [72], we sought to confirm taxon classification using both principal component analysis (PCA) and neighbour-joining trees (NJT). To validate taxon assignments, we analyzed our samples together with Kenyan *Anopheles gambiae s. l.* samples from the MalariaGEN Vector Observatory [15, 53, 73, 74]. Samples represented known closely related and morphologically indistinct taxa from East Africa, including *An. gambiae*, *An. arabiensis*, *An. coluzzii,* and the *Pwani* molecular form [27]. The PCA and NJT revealed five distinct groups representing individuals of *An*. *arabiensis*, *An. coluzzii*, *Pwani* molecular form*,* and two groups of *An. gambiae*. The two groups of *An. gambiae* include samples from coastal Kilifi in Kenya and the inland populations of Busia and Turkana in Kenya, reflecting the restricted gene flow previously observed between coastal and inland populations in East Africa [53] (Fig. 2). All Ethiopian *An*. *gambiae s.s.* clustered with inland East African *An. gambiae,* confirming their taxonomic status.

### Target-site resistance

**Fig. 3:**
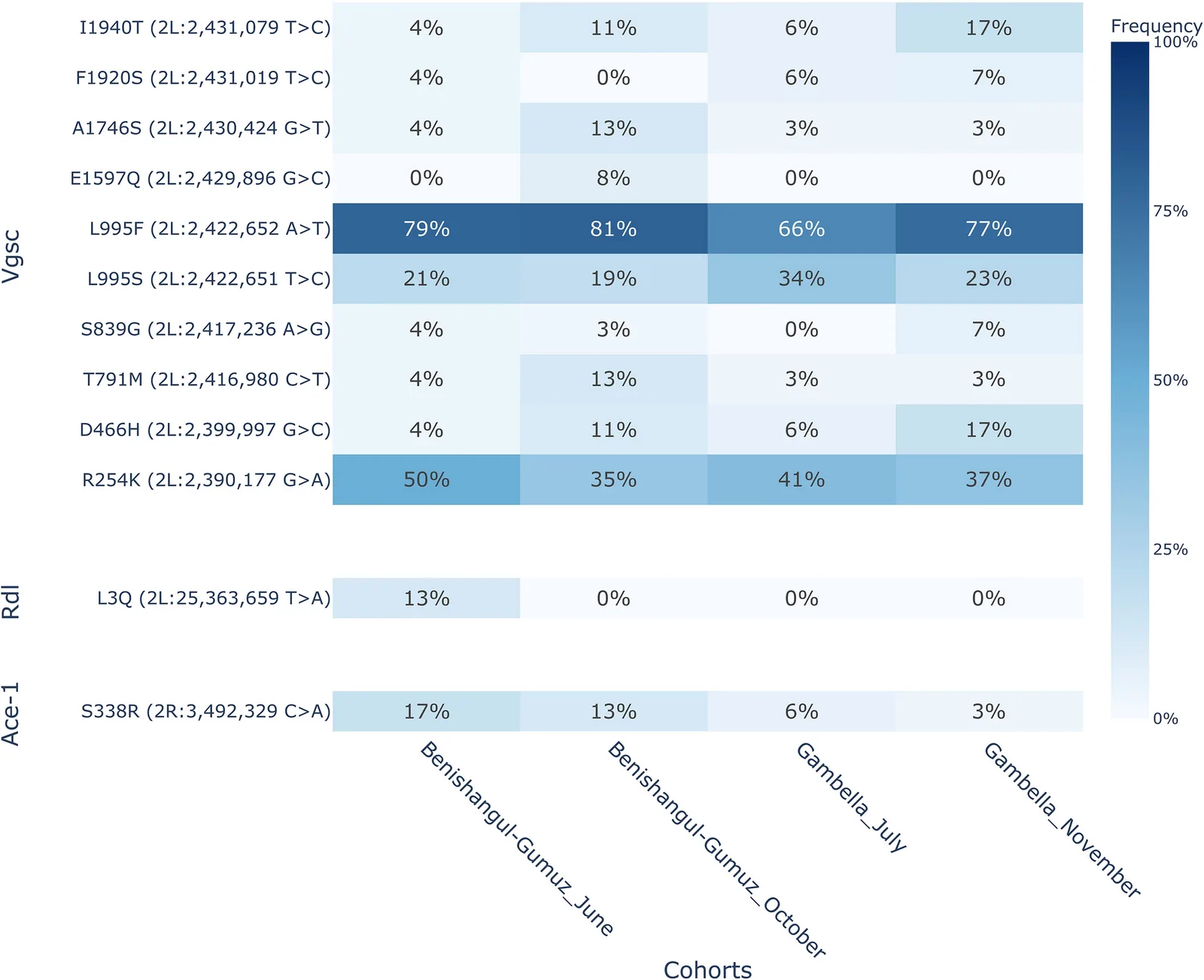
A heatmap plot showing the frequency of amino acid substitutions at the *Vgsc*, *Ace-1*, and *Rdl* genes in *Anopheles gambiae* sensu stricto from Western Ethiopia. The x-axis labels indicate the sampling cohorts, grouped by the administrative region (Benishangul-Gumuz and Gambella) and month of collection in 2023 (June, July, October, and November)

Genes coding for insecticide targets, including pyrethroids, organophosphates, and organochlorines, were analyzed for the presence of associated single-nucleotide polymorphisms (SNPs) from western Ethiopia. The voltage-gated sodium channel (*Vgsc*), acetylcholinesterase-1 (*Ace-1*), and gamma-aminobutyric acid (GABA)-gated chloride channel (*Rdl*) genes were investigated. The *Rdl* gene encodes a subunit of the insect GABA-gated chloride channel, which was the primary target of the organochlorine insecticide dieldrin. Although dieldrin is no longer in operational use due to its persistence and toxicity, studying *Rdl* remains highly relevant as it is also the target of insecticides such as fipronil and can provide cross resistance to neonicotinoids and pyrethroids. High frequencies of *Vgsc*-L995F, known to confer pyrethroid resistance in *Anopheles gambiae* and *Anopheles arabiensis* [75], were found in all cohorts from Western Ethiopia, but were higher in Benishangul-Gumuz (79–81%) than in Gambella (66–77%). Furthermore, a slight increase in frequencies was observed in both cohorts moving from the minor (June and July) to the major malaria transmission season (October and November) (Fig. 3). Another substitution associated with pyrethroid resistance [75], *Vgsc-*L995F (kdr-east), was observed at moderate frequencies (19–34%) in all cohorts. Between the two survey time points, the frequency of *Vgsc-*L995S decreased while *Vgsc-*L995F increased in both Benishangul-Gumuz and Gambella. We did not find the *Ace-1 *G119S [76] or *Rdl*-A296S or A296G substitutions [77] linked to insecticide resistance in *An. gambiae* from Ethiopia (Fig. 3).

### Metabolic resistance

**Fig. 4:**
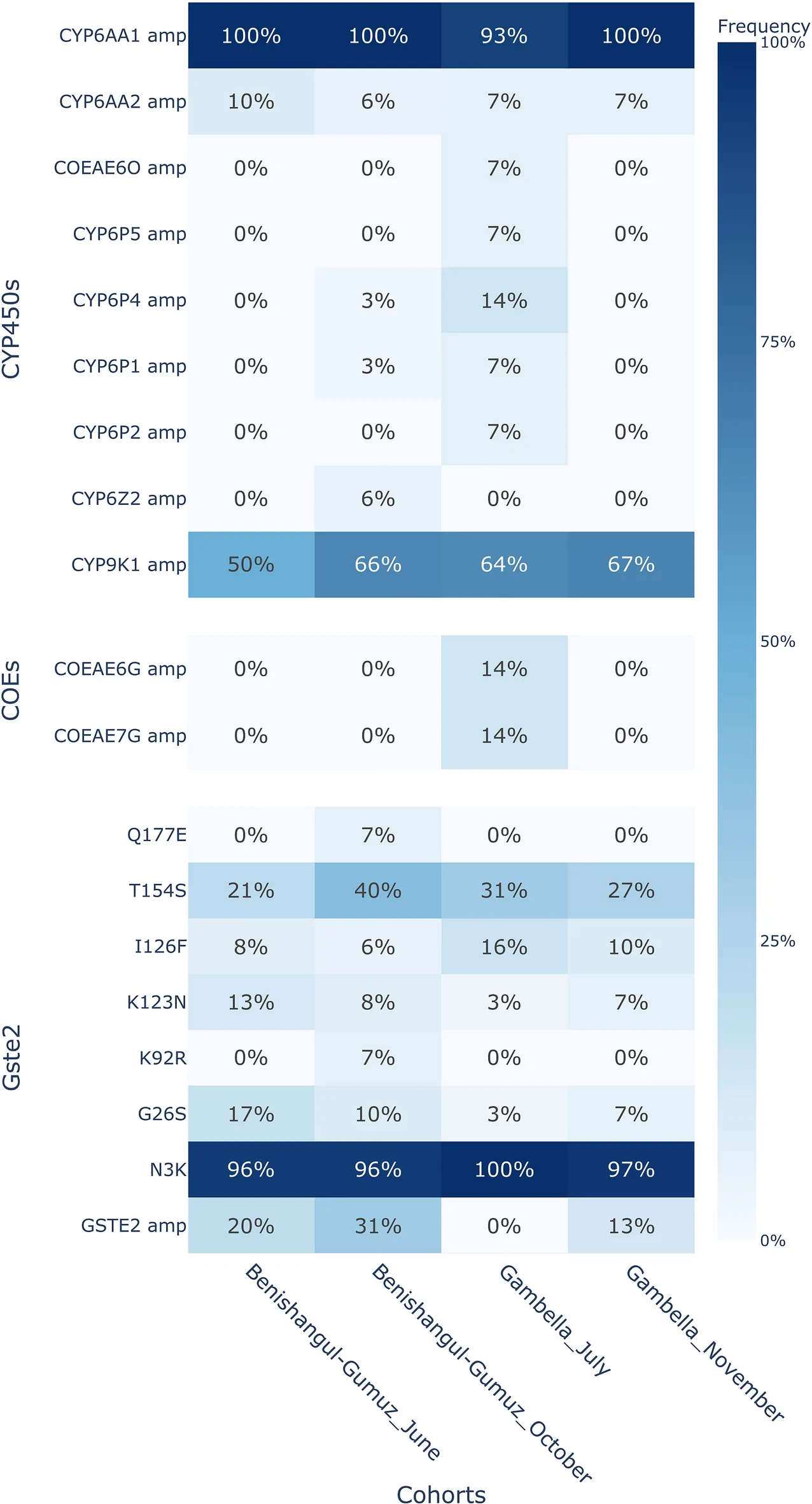
A heatmap plot showing the frequency of copy number variants (CNVs) at Cytochrome P450 genes (*Cyp450s*), including *Cyp6aa/p* and *Cyp9k1*, carboxylesterase genes (COEs), including *Coeae2-7 g,* and both CNVs and substitutions at the *Gste2* locus in *Anopheles gambiae s. s.* across minor (April-June) and major (September–November) malaria transmission seasons in the Benishangul-Gumuz and Gambella regions in western Ethiopia

**Fig. 5:**
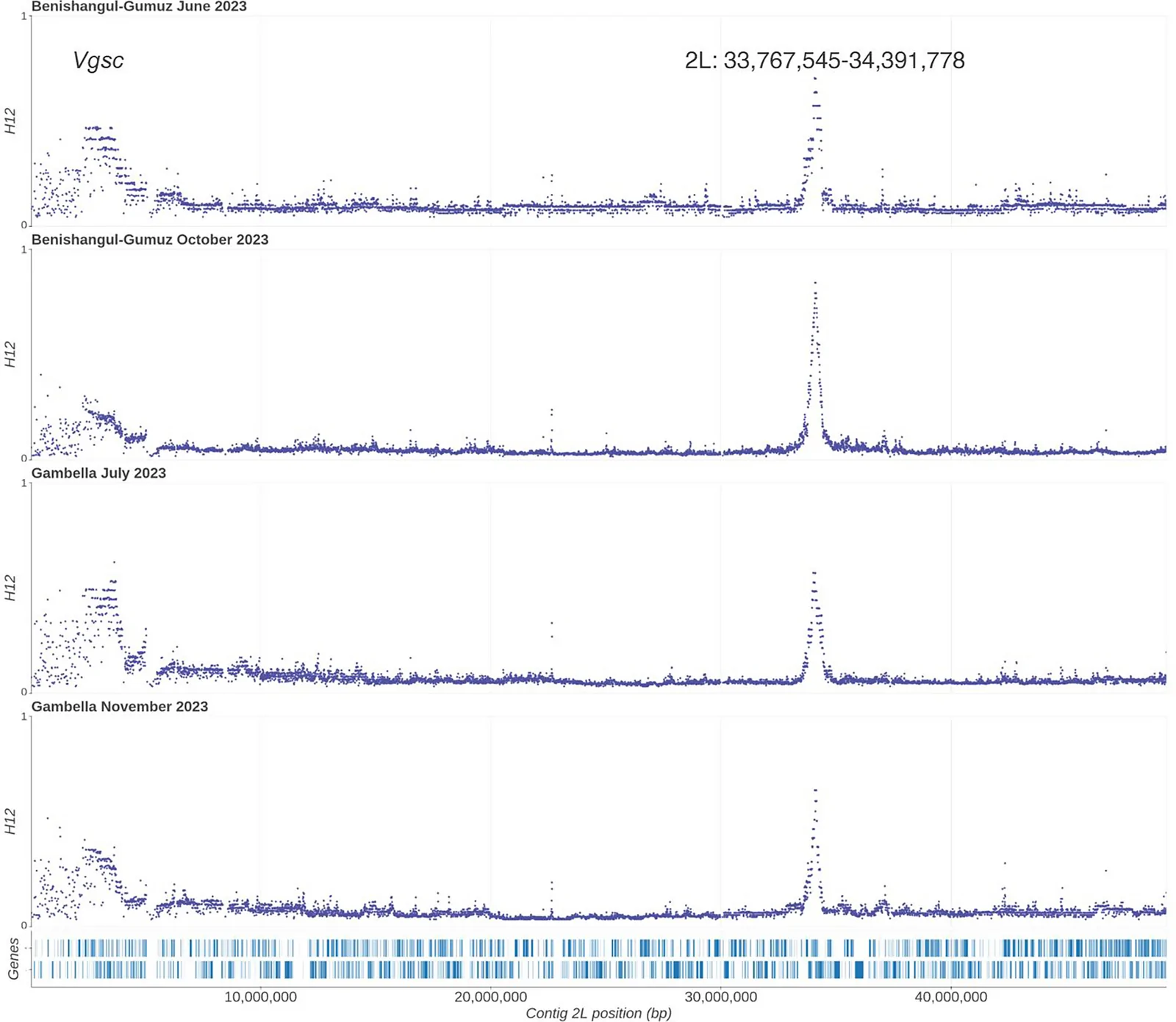
Plots of genome-wide selection scans of the H12 statistic across the 2L chromosome arm for each population cohort. Regions with elevated H12 values indicate a recent signal of selection and are annotated on the plot

We investigated metabolic gene clusters associated with *Anopheles* insecticide resistance, including glutathione-S-transferases (*Gste2*) [30], the cytochrome P450 gene clusters (*Cyp6aa/p, Cyp6m2,* and *Cyp9k1*) [78], and the carboxylesterase gene clusters *(Coeae2f* and *Coeae2-7 g*) [23, 79]. For each gene, we evaluated the frequency of individuals with at least one copy number variant (CNV) (Fig. 4). Amino acid substitution frequencies were also analyzed for *Gste2* since the L119V substitution has been functionally validated to confer permethrin (pyrethroid class) target-site resistance [80]. However, we did not observe the *Gste2*-L119V substitution in *An. gambiae* from Ethiopia, we found CNV amplifications that were higher in Benishangul-Gumuz (20–31%) than in Gambella (0–13%). Amplification frequencies were also higher in both locations during the minor malaria transmission season in June and July (Fig. 5). CNV amplifications at *Cyp6aa1* were fixed across all cohorts. The amplification of a relatively low number of individuals (6–10%) also extended across the *Cyp6aa2* gene in all cohorts and also extended further from *Cyp6aa1* to *Cyp6p2* in *An. gambiae* from Gambella in July. CNVs at *Cyp9k1* were present at a high frequency (50–67%) and increased during the major malaria transmission season in both locations. This increase was only 3% in Gambella, but more pronounced in Benishangul-Gumuz with a 16% increase. Amplifications were present at the *Coeae2-7* g gene cluster at a 14% frequency, but this was only observed in Gambella in July, suggesting their presence may be temporary. We did not observe CNVs at the *Coeae2f* locus for all cohorts (Fig. 4).

### Selection scans

To detect signals of recent positive selection across the genomes, we calculated Garud’s haplotype homozygosity H12 statistic in 1000–2500 base pair windows across all chromosome arms [81]. One prominent selection peak spanned the region 2L:33,767,545–34,391,778 which encompasses a variety of genes, including acetyl-CoA synthetase (AGAP006569), *UDP-glucose 6-dehydrogenase* (AGAP006532), and *outer segment 1* (*Oseg1*, AGAP006535) (Table S3).

We also observed a selection peak in all cohorts at the *Vgsc* on 2L (Fig. 5), the *Cyp6aa/p* gene cluster on 2R, and *Gste2* on 3R, as well as an indistinct peak at *Cyp9k1* on the X chromosome (Figure S2 a–e), which concurs with our findings of either substitutions or CNVs associated with insecticide resistance at these loci (Fig. 4).

## Discussion

Our taxonomic analysis of whole-genome sequences of *Anopheles gambiae *sensu lato mosquitoes confirms the presence of *Anopheles gambiae s. s.* in Ethiopia, consistent with an earlier report in south-central Ethiopia [82]. We have revealed that *An. gambiae* s. s. is found in western Ethiopia, where it was previously unreported. Our findings raise an important question about whether the presence of *An. gambiae s. s.,* a highly efficient, anthropophilic vector capable of maintaining transmission even at low mosquito densities, contributes to the persistent, year-round malaria burden in western Ethiopia, where *An. arabiensis* is considered the major malaria vector [8, 83]. Our findings underscore the importance of revisiting the geographical distribution of malaria vectors in Ethiopia, where numerous studies have investigated *Anopheles* populations, but only one reported *An. gambiae s. s.* in south-central Ethiopia based on a traditional molecular assay [82]. In addition, we have revealed that *An. gambiae* in Ethiopia have evidence of emerging molecular insecticide resistance mechanisms under selection, similar to those reported from elsewhere in East Africa [53, 84].

*An. gambiae* in western Ethiopia carried several metabolic resistance markers, with CNV frequencies similar in Benishangul-Gumuz and Gambella. For example, we observed amplifications at the *Cyp6aa*/*p* locus at a very high frequency in our samples from western Ethiopia. Consistent with previous reports from East African *An. gambiae* populations [53, 85], these amplifications included the *Cyp6aa1* locus. Duplication of *Cyp6aa1* has been widely and increasingly documented as strongly associated with metabolic resistance to pyrethroids and other insecticides [78, 86–88]. Our findings provide additional evidence that *Cyp6aa1* CNV amplifications are widespread in East Africa. Furthermore, amplification frequencies for *Cyp9k1,* associated with enhanced metabolic detoxification of pyrethroids [89, 90], were generally similar across sites, although the frequency was lower in the Benishangul-Gumuz cohort from June. Together, findings suggest a similar selective pressure impacting these loci across western Ethiopia, linked to insecticide exposure across the region. In support of this notion, both regions implement similar vector control interventions, including LLINs and IRS, which use both pyrethroids and carbamates [91–93]. Additionally, the two sampling sites are approximately 500 km apart, and recent genomic studies have reported extensive gene flow among *An. gambiae* populations across large geographical distances, including ecological zones and national borders [94, 95], *An. gambiae* 1000 Genomes Consortium 2017). Therefore, it is likely that gene flow and the exchange of adaptive alleles occur between the two locations in western Ethiopia, although this requires further study on the population structure and connectivity of *An. gambiae* across the country.

Although we observed some similarities, the frequency of metabolic resistance loci such as *Gste* and the target-site resistance gene *Vgsc* differed on a localized scale. For example, we observed that *Gste2* amplifications were notably higher in Benishangul-Gumuz compared to Gambella, suggesting a differential selection pressure or different evolutionary trajectory for this locus. Although both regions implement similar malaria control strategies, the widespread use of agrochemicals in agricultural communities may contribute to localized environmental exposure [96]. Increasingly, studies on the geographical distribution of insecticide resistance markers have cited agricultural chemical use as a potential influencing factor [97]. Although information on local insecticide and pesticide use in Ethiopia is generally lacking, a recent environmental assessment by the Ethiopian Environmental Protection Authority (EEPA) further highlights escalating land degradation and vegetation stress in Benishangul-Gumuz, driven in part by slash-and-burn agriculture and chemical inputs, which may contribute to off-target selection pressure on *Anopheles* populations [98]. Although we found that the *Vgsc*-L995F substitution impacting the target-site of pyrethroids was detected at high frequencies in both sites, we found that frequencies were also slightly elevated in Benishangul-Gumuz. However, this difference was minor, and confirmation requires further longitudinal sampling across multiple years since both sites are expected to receive sustained pyrethroid pressure resulting from their widespread use in vector control [99–101]. Pairing agricultural chemical use surveys or soil testing with genomic data and/or susceptibility testing would help clarify the role of agriculture in contributing to insecticide resistance in western Ethiopia.

Although we lack phenotypic data to directly confirm insecticide resistance phenotypes in *An. gambiae* populations from Ethiopia, genomic markers provide a useful proxy for resistance status. We observed seasonal shifts in allele frequencies at key pyrethroid resistance loci, including *Vgsc*-L995F and *Cyp9k1*. *Vgsc*-L995F showed a slight increase across the minor (June-July) and major (October–November) transmission seasons in all cohorts, in contrast to *Vgsc*-L995S, which showed a slight decrease. This observation is consistent with previous reports indicating that *Vgsc*-L995F and *Vgsc*-L995S are typically found on distinct haplotypes and have different fitness effects, which appear context-dependent [99]. While *Vgsc*-L995F has been associated with enhanced survival under insecticide pressure, it may also incur fitness costs in the absence of insecticides, potentially influencing its frequency in natural populations [61, 99, 102]. Although we have limited temporal resolution, our findings suggest that *Vgsc*-L995F tends to increase in frequency relative to *Vgsc*-L995S in wild populations during the major malaria transmission season, when insecticide use for malaria control is expected to be higher [103, 104], while appearing less prevalent during the minor transmission season, possibly reflecting an associated fitness cost. Along with shifts in insecticide use for malaria control, the higher frequencies of both *Vgsc*-L995F and *Cyp9k1* amplifications during the major transmission season could result from increased use of OP and pyrethroid-based agrochemicals applied during the major agricultural season, which coincides with the peak malaria transmission period (September to November) [96]. During this time, irrigated and flood-prone agricultural zones expand following the major rainy season, increasing the likelihood of mosquito larval exposure to sub-lethal doses of pesticides. Additionally, vector control interventions, such as IRS and ITNs, are typically scaled up during this period to curb transmission [103, 104]. Consistent with this seasonal intensification of chemical exposure, we observed a notable increase in *Gste2* amplification frequencies linked to DDT, organophosphate, and pyrethroid resistance [30, 100, 105, 106] during the major transmission season in western Ethiopia. Interestingly, we further observed a 19% increase in *Gste2*-T154S*,* a substitution implicated in the detoxification of pyrethroids [107], although this was restricted to Benishangul-Gumuz. However, to date, this variant has not been functionally validated for its role in insecticide resistance, and its phenotypic impact remains uncertain. Taken together, this temporal and regional difference raises the hypothesis that localized selection pressures, potentially driven by pyrethroid exposure, are shaping resistance allele dynamics in Ethiopian *An. gambiae* populations.

We observed an H12 selection scan peak at the genomic region spanning 2L:33,767,545–34,391,778 centred on acetyl-CoA synthetase (AGAP006569), a metabolic gene implicated in energy regulation and detoxification [108]. Our detection of a strong selection signature at both sampled locations for this locus aligns with transcriptomic and genomic surveillance by Nagi et al. (2025), who identified AGAP006569 as part of a broader group of metabolic genes potentially under selection in *An. gambiae*, particularly in populations from Kenya exposed to mixed agrochemical and public health insecticides. Furthermore, this gene’s upregulation has been linked to enhanced survival under pressure from pyrethroids and organophosphates [109, 110], suggesting it as a candidate for functional validation. Other nearby genes like *UDP-glucose 6-dehydrogenase* (AGAP006532) and *outer segment 1* (*Oseg1*, AGAP006535) were previously identified under a selection peak in Kenyan populations at position 2L: 33.910,602–33.986,817 and also potentially have an indirect role in insecticide resistance through cuticular modifications or sensory adaptation [79, 111]. However, these genes did not fall under the peak we observed. Together with these studies, our findings indicate that selection on the 2L region is impacting *An. gambiae* across the wider East African region, emphasizing its growing significance as a locus of concern for vector control efforts.

## Conclusions

This study presents the first whole-genome sequencing-based characterization of *Anopheles gambiae* in western Ethiopia, enabling high-resolution analysis of species identity and molecular insecticide resistance architecture, generating insights that can inform malaria control in the region. The confirmed presence of *An. gambiae s. s.*, a species with high vectoral capacity across East Africa, raises important concerns about its potential role in sustaining transmission, particularly in ecologically permissive zones of Ethiopia, where its distribution has been poorly characterized. To better characterize its presence in Ethiopia, the entomological surveillance of *An. gambiae* should be incorporated into routine regional monitoring. Our finding that *An. gambiae s. s.* in Ethiopia have a high frequency of both *Vgsc-*﻿L995F target site and metabolic resistance-associated variants, including copy number amplifications at *Cyp6aa/p, Cyp9k1,* and *Gste2*, suggests that populations may be largely refractory to pyrethroids and potentially moderately resistant to OPs, the cornerstone insecticides of current vector control programmes. Although confirmation with bioassay is required, it is likely this resistance profile threatens the efficacy of both indoor residual spraying (IRS) and long-lasting insecticidal nets (LLINs), necessitating the development of species-specific and resistance-informed intervention strategies. The suggestion of seasonal fluctuations in *An. gambiae* molecular insecticide resistance marker frequencies underscore the critical importance of temporally resolved monitoring. Therefore, continuous monitoring in line with the aims and logistical capabilities of the national vector control programme would be valuable, as would the use of both bioassays and whole-genome analysis in assessing the effectiveness of newly introduced intervention strategies. Importantly, this study identifies a newly emerging signal of selection across multiple loci spanning the region 2L:33,767,545–34,391,778. Our findings highlight its importance as an emerging signal of selection in East Africa and underscore the value of population genomics for detecting adaptive changes beyond canonical markers. Priority next steps include (i) the routine monitoring of both taxonomic and insecticide resistance molecular markers into national vector management campaigns (ii) functional assays for acetyl-CoA synthetase and its neighbouring genes.

## Supplementary Information

Supplementary material 1. Figure S1. Plot of taxon designation based on ancestry-informative markers (AIM) distinguishing **a** *Anopheles gambiae* from *Anopheles coluzzii* and, in comparison, from the *Anopheles pwani* molecular form, which has a higher proportion of intermediate alleles, and **b** *Anopheles gambiae*/*coluzzii *from *Anopheles* *arabiensis*. In the legend: the “colu/colu” shows the AIM genotype of *Anopheles coluzzii* against itself; “gamb/colu” shows the AIM genotype of *An. gambiae* against *An. coluzzii*; “gamb/gamb” shows the AIM genotype of *An. gambiae* against itself.

Supplementary material 2. Figure S2. Genome-wide selection scans with peaks indicating selective sweeps: **a** on the 2R contig (*Cyp6aa/p *gene cluster), **b** 3R (*Gste2*), **c** 2L contig (*Vgsc*), **d** contig X (*Cyp9k1*) and **e** 3L contig/chromosome arms using the H12 statistic.

Supplementary Material 3. Table S1. Putative genes showing increased H12 values (peaks) in both Gambella and Benishangul-Gumuz *Anopheles gambiae *cohorts.

Supplementary Material 4. Table S2. Sample sets and accession numbers for *Anopheles gambiae* sequence data.

## Data Availability

The sequences of the samples identified in this study were submitted to the European Nucleotide Archive (ENA; accession numbers in Table S2).

## Abbreviations

ACE-1: *A**cetylcholinesterase*-1
CNV: Copy number variants
*Cyp450s*: Cytochrome P450 monooxygenase system
ENA: European Nucleotide Archive
GABA: Gamma-aminobutyric acid
Gste: Glutathione S-transferase epsilon class genes
GWSS: Genome-wide selection scan
IRS: Indoor residual spraying
LLINs: Long-lasting insecticidal nets
OP: Organophosphates
Rdl: Resistance to dieldrin
SNPs: Single-nucleotide polymorphisms
Vgsc: Voltage-gated sodium channel
