# Supplementary material for: Genomic analysis reveals selection pressure and the emergence of molecular insecticide resistance in the malaria vector, Anopheles gambiae, from Western Ethiopia
Source: Parasit Vectors. 2026 Aug 5;19:471. doi: 10.1186/s13071-026-07582-z (PMC13726317; doi:10.1186/s13071-026-07582-z)
Supplement: Supplementary file 3 [file 13071_2026_7582_MOESM3_ESM.docx]

| **Gene ID** | **Contig** | **Start** | **End** | **Name** | **Description** |
| --- | --- | --- | --- | --- | --- |
| AGAP006517 | 2L | 33,767,545 | 33,773,835 | Gryzun | protein-coding [gene](http://www.sequenceontology.org/browser/obob.cgi) |
| AGAP006518 | 2L | 33,776,282 | 33,777,612 | charged multivesicular body protein 1 | protein-coding [gene](http://www.sequenceontology.org/browser/obob.cgi) |
| AGAP006532 | 2L | 33,939,186 | 33,946,087 | UDP-glucose 6-dehydrogenase | protein-coding [gene](http://www.sequenceontology.org/browser/obob.cgi) |
| AGAP006535 | 2L | 33,963,337 | 33,969,843 | Oseg1 | outer segment 1 |
| AGAP006533 | 2L | 33,948,755 | 33,950,031 | surfeit locus 1 family protein | protein-coding [gene](http://www.sequenceontology.org/browser/obob.cgi) |
| AGAP006569 | 2L | 34,158,499 | 34,168,017 | acetyl-CoA synthetase | protein-coding [gene](http://www.sequenceontology.org/browser/obob.cgi) |
| AGAP006570 | 2L | 34,168,871 | 34,170,407 | myo-inositol-1(or 4)-monophosphatase | protein-coding [gene](http://www.sequenceontology.org/browser/obob.cgi) |
| AGAP006571 | 2L | 34,170,872 | 34,203,002 | nuclear receptor subfamily 1 group D member 3 | protein-coding [gene](http://www.sequenceontology.org/browser/obob.cgi) |
| AGAP006590 | 2L | 34,288,135 | 34,391,778 | T-lymphoma invasion and metastasis-inducing protein 1 | protein-coding [gene](http://www.sequenceontology.org/browser/obob.cgi) |
