# Supplementary material for: Genomic analysis reveals selection pressure and the emergence of molecular insecticide resistance in the malaria vector, Anopheles gambiae, from Western Ethiopia
Source: Parasit Vectors. 2026 Aug 5;19:471. doi: 10.1186/s13071-026-07582-z (PMC13726317; doi:10.1186/s13071-026-07582-z)
Supplement: Supplementary file 4 [file 13071_2026_7582_MOESM4_ESM.docx]

| **Sample sets** | **Accession numbers** |
| --- | --- |
| 1324-VO-ET-GOLASSA-VMF00257 | ERR12314211-ERR12314243 |
| 1324-VO-ET-GOLASSA-VMF00257 | ERR12326237-ERR12326277 |
| 1324-VO-ET-GOLASSA-VMF00275 | ERR12983154-ERR12983193 |
| 1324-VO-ET-GOLASSA-VMF00275 | ERR13101381-ERR13101432 |
